# Role of HDAC9-FoxO1 Axis in the Transcriptional Program Associated with Hepatic Gluconeogenesis

**DOI:** 10.1038/s41598-017-06328-3

**Published:** 2017-07-21

**Authors:** Jizheng Chen, Zhilei Zhang, Ning Wang, Min Guo, Xiumei Chi, Yu Pan, Jing Jiang, Junqi Niu, Sulaiman Ksimu, John Zhong Li, Xinwen Chen, Qian Wang

**Affiliations:** 1 Jiangsu Province Key Lab of Human Functional Genomics, Department of Biochemistry and Molecular Biology, Nanjing Medical University, Nanjing, 210029 China; 2 State Key Lab of Virology, Wuhan Institute of Virology, Chinese Academy of Sciences, Wuhan, 430071 China; 3 State Key Laboratory of Natural Medicines, School of Life Science and Technology, China Pharmaceutical University, Nanjing, Jiangsu 210009 China; 4 Department of Hepatology, The First Hospital of Jilin University, Changchun, 130021 China; 5 The Center for Technology and Education, The first Affiliated Hospital of Xinjiang Medical University, Urumchi, 830054 China

## Abstract

Histone deacetylase 9 (HDAC9) regulates hepatic gluconeogenesis by deacetylating Forkhead box O 1 (FoxO1). HDAC9 upregulation is involved in hepatitis C virus (HCV)-associated exaggerated gluconeogenesis. Herein, we found in addition to FoxO1, HDAC9 also regulates other gluconeogenic transcription factors, including peroxisomeproliferator-activated receptor-γ coactivator-1α (PGC-1α), cyclic AMP-responsive element-binding protein (CREB), and glucocorticoid receptor (GR). Unlike FoxO1, which is regulated by post-translational modification responses to HDAC9, HDAC9 regulates PGC-1α, CREB and GR by altering gene expression. Similar to PGC-1α, CREB and GR were found to be novel regulatory targets of FoxO1 by examination of the FoxO1 binding site in their promoter. PGC-1α, CREB and GR were upregulated in response to HDAC9 via FoxO1 deacetylation. These findings indicate that HDAC9-FoxO1 signalling contributes to gluconeogenesis by modulating the expression of gluconeogenic transcription factors. In particular, metabolic profiling demonstrated a clear shift towards gluconeogenesis metabolism, and HDAC9-FoxO1 signalling can be strongly induced to upregulate gluconeogenic transcription factors following HCV infection. The positive correlation between HDAC9 and gluconeogenic transcription factor expression levels in the livers of both HCV-infected patients and normal individuals further emphasizes the clinical relevance of these results. Thus, HDAC9-FoxO1 signalling axis is involved in regulating gluconeogenic transcription factors, gluconeogenesis, and HCV-induced type 2 diabetes.

## Introduction

Glucose homeostasis is maintained through tight regulation of glucose production in the liver and glucose uptake in the peripheral tissues, particularly skeletal muscle and adipose tissue. Hepatocytes play an important role in maintaining plasma glucose homeostasis by balancing hepatic glucose production and utilization via the gluconeogenic and glycolytic pathways, respectively. Excessive hepatic gluconeogenesis and glucose production are important factors involved in the development of hyperglycaemia in patients with metabolic syndromes and type 2 diabetes^1, 2^.

Gluconeogenesis is largely regulated at the transcriptional level by control of the rate-limiting enzymes phosphoenolpyruvate carboxykinase (PEPCK) and glucose-6-phosphatase (G6Pase). Expression of these enzymes is controlled via hormonal modulation of transcription factors and coactivatorssuch as the forkhead box O (FoxO) proteins, peroxisome proliferator-activated receptor-γ (PPAR-γ) coactivator-1α (PGC-1α), cyclic AMP (cAMP)-responsive element (CRE)-binding protein (CREB), and glucocorticoid receptor (GR)^3–6^.

PGC-1α is a master regulatory co-activator of the gluconeogenic transcription programme induced upon fasting. Transcriptional regulation of PGC-1α by CREB is a common pattern in the metabolic adaption of the liver to gluconeogenic status in response to glucagon and glucocorticoids^7^. Besides, signalling via protein kinase B (PKB) to Forkhead box O 1 (FoxO1), a FoxO family transcription factor, also partly account for the effect of insulin in regulating PGC-1α promoter activity via the insulin response sequence (IRS)^8^. Alternative splicing or transcription initiation represents another mode of regulation. A recent study has characterized a novel human liver-specific PGC-1α (L-PGC-1α) transcript which results from alternative promoter usage^9^.

Recent studies have demonstrated that the regulation of glucose homeostasis is associated with epigenetic mechanisms^10^. Post-translational modifications by acetylation, phosphorylation, methylation, and ubiquitination play important roles in gene transcription. In particular, the acetylation of histone and non-histone proteins provides a key mechanism for controlling cellular signalling and gene expression. The histone deacetylases (HDACs) regulate this acetylation of histone and transcriptional factors involved in glucose homeostasis, thereby playing a central role in the regulation of glucose metabolism^11^. Among them, class IIa HDACs (HDAC4, 5, 7) are hormone-activated regulators of FoxO and mammalian glucose homeostasis^12^. Our recent studies have indicated that HDAC9, another class IIa HDAC, regulates hepatic gluconeogenesis via deacetylation of FoxO1 together with HDAC3^13^.

Epidemiological and experimental data indicate that hepatitis C virus (HCV) infection is involved in the development of diabetes, especially T2DM^14^. There is growing evidence to support the hypothesis that chronic HCV infection is a risk factor for developing T2DM. HCV infection *per se* is associated with insulin resistance in the target pathways of endogenous glucose production and total body glucose disposal^15^. We have reported that chronic HCV infection may induce HDAC9 expression, resulting in an exaggerated gluconeogenic response likely mediated by FoxO1 transcription factor activity, directly predisposing the host to abnormal glucose metabolism^13^.

In this report, we show that FoxO1-binding sites in the upstream regions of CREB and GR mediate transcriptional responsiveness to FoxO1. HDAC9 upregulates the expression of PGC-1α, CREB and GR via deacetylation of FoxO1, implicating the HDAC9-FoxO1 signalling pathway as a key regulator of gluconeogenic genes, transcriptional factors, and gluconeogenesis metabolism in hepatocytes. Using metabolomics assays, we demonstrate a clear shift towards gluconeogenesis metabolism following HCV infection and show that the HDAC9-FoxO1 signalling axis is essential for HCV-induced upregulation of gluconeogenic transcriptional factors and exaggeration of gluconeogenesis. Our findings provide novel insight into the roles of key metabolic regulators in hepatic cells as well as clues to the mechanisms underlying the development of HCV-induced glucose abnormality.

## Results

### HCV infection redirects cellular metabolism towards gluconeogenesis

**Figure 1:**
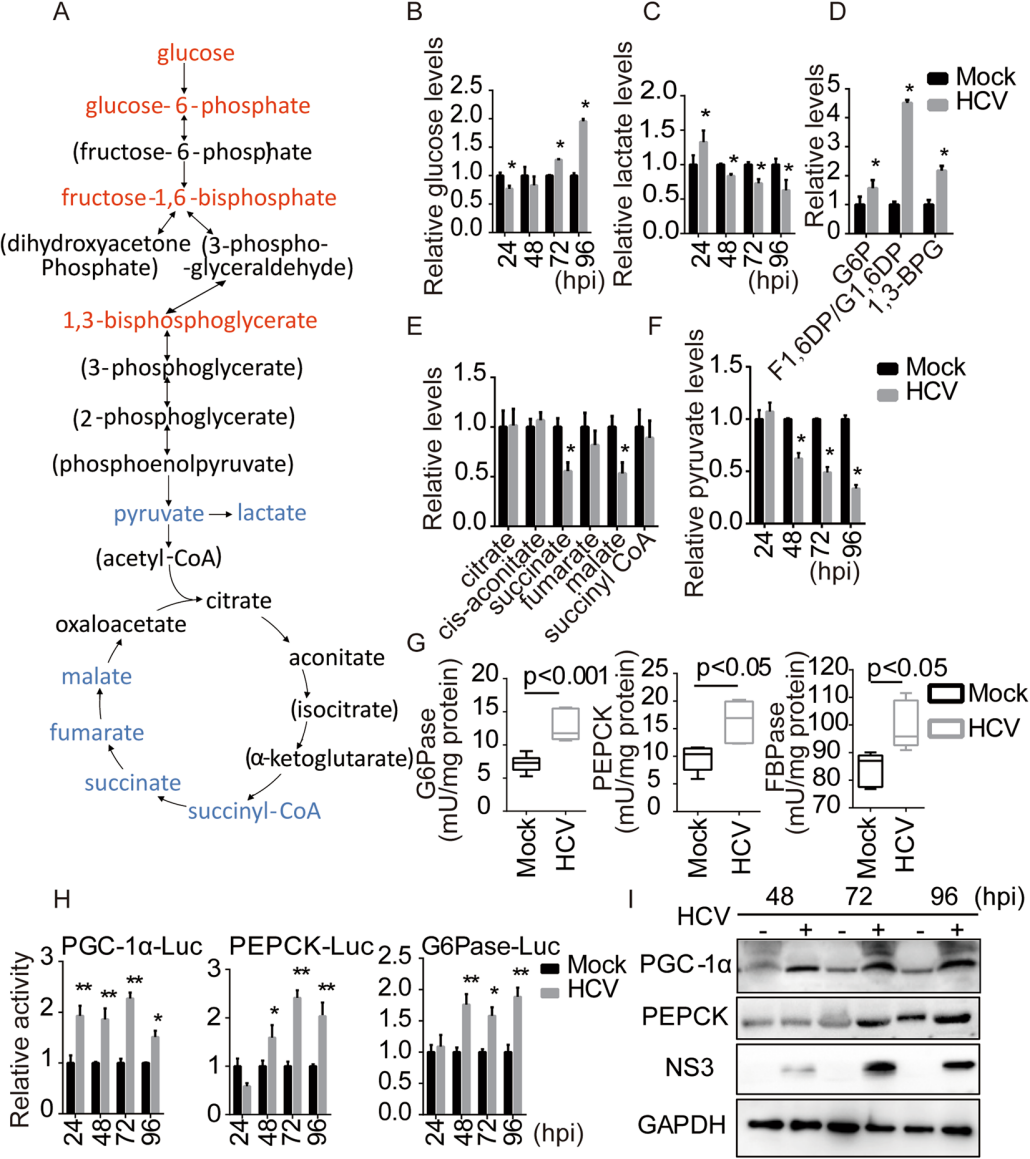
HCV infection redirects cellular metabolism towards gluconeogenesis. (**A**) Schematic illustrating the metabolites that are increased (red) or decreased (blue) in HCV-infected-HuH7 cells at 0.1 MOI and 96 hpi compared to mock-infected cells. Metabolites in parentheses were not measured. Levels of glucose (**B**), lactate (**C**), and pyruvate (**F**) at the indicated time points. Levels of glycolytic intermediates (**D**) and TCA cycle intermediates (**E**) at 96 hpi. Box plot diagram showing PEPCK, G6Pase and FBPase enzymatic activity (**G**) in mock- or HCV-infected-cells at 0.1 MOI and 96 hpi. The promoter activity of PGC-1α, PEPCK, G6Pase (**H**), and related protein (**I**) levels in mock- or HCV(0.1 MOI)-infected-cells at the indicated time points. Data are represented as the mean ± SEM for triplicate experiments.

Previous studies have indicated that HCV promotes hepatic gluconeogenesis^13, 16^. To gain insight into the metabolic signature associated with this phenomenon, we examined the influence of HCV infection on the metabolites involved in carbohydrate metabolism and energy pathways from HCV-infected cells using NMR analysis (Supplementary Table S1). Increases in the amount of the viral RNA between 24 and 96 h post infection (hpi) indicated effective replication of the virus (Supplementary Fig. S1A). The metabolic profile of HCV-infected cells demonstrated a clear shift towards gluconeogenesis metabolism compared with mock-infected counterparts between 24 and 96 hpi (Fig. 1A). In HCV-infected cells, intermediates of glycolysis were elevated (Supplementary Fig. S1B), whereas TCA cycle metabolites were stable at 24 hpi (data not shown). Consistent with this pattern, HCV-infected cells showed lower levels of intracellular glucose (Fig. 1B) and produced more lactate (Fig. 1C). However, levels of glycolysis intermediates were continually elevated (Fig. 1D), whereas TCA cycle metabolite levels were significantly reduced at 96 hpi (Fig. 1E). Most steps in gluconeogenesis are the reverse of those in glycolysis. Thus, to confirm that the observed metabolite patterns reflected an increase in gluconeogenesis, we further quantified glucose production and lactate levels. As expected, HCV-infected cells exhibited decreased pyruvate concentrations and consumed more lactate, accompanied by higher levels of intracellular glucose compared to control cells over time (Fig. 1B,C,F). Consistent with the metabolite profiles of an induced shift to gluconeogenesis, the enzymatic activities of PEPCK, G6Pase, and FBPase were significantly elevated in HCV-infected cells compared to those in mock-infected cells at 96 hpi (Fig. 1G). These results are in agreement with a previous report of elevated transcriptional levels of rate-limiting gluconeogenic enzymes (data not shown)^13^.

PGC-1α is an important regulatory co-activator of gluconeogenic gene transcription. Previous studies have shown that PGC-1α is upregulated following HCV infection^17, 18^. To investigate this phenomenon in detail, the promoter activity of PGC-1α was analysed and was found to be increased in HCV-infected cells (Fig. 1H) in parallel with elevated HCV NS3 and PGC-1α protein levels (Fig. 1I), similar to the effect observed in HCV subgenomic replicons (Supplementary Fig. S1C,D). Meanwhile, the promoter activity of key enzymes involved in gluconeogenesis (Fig. 1H, Supplementary Fig. S1C) and glucose production (data not shown) were upregulated in HCV-infected cells. This effect was reversed by treatment with 2mAde (an HCV inhibitor that targets the viral NS5B protein), in agreement with previous results (Supplementary Fig. S1E,F). Moreover, PGC-1α silencing significantly attenuated the promoter activity and expression levels of PEPCK and G6Pase as well as glucose production both in HuH7 cells and HCV-infected hepatocytes (Supplementary Fig. S1G–I). We also detected another gluconeogenic transcription factor, CREB, but found that HCV infection did not significantly upregulate its expression (data not shown).

These data suggest that HCV infection redirects cellular metabolism towards gluconeogenesis during a late stage of infection and that HCV-induced upregulation of PGC-1α plays a role in regulating gluconeogenesis in hepatic cells and in HCV-promoted gluconeogenesis.

### Positive correlation between HDAC9 and PGC-1α in HCV-infected patients

**Figure 2:**
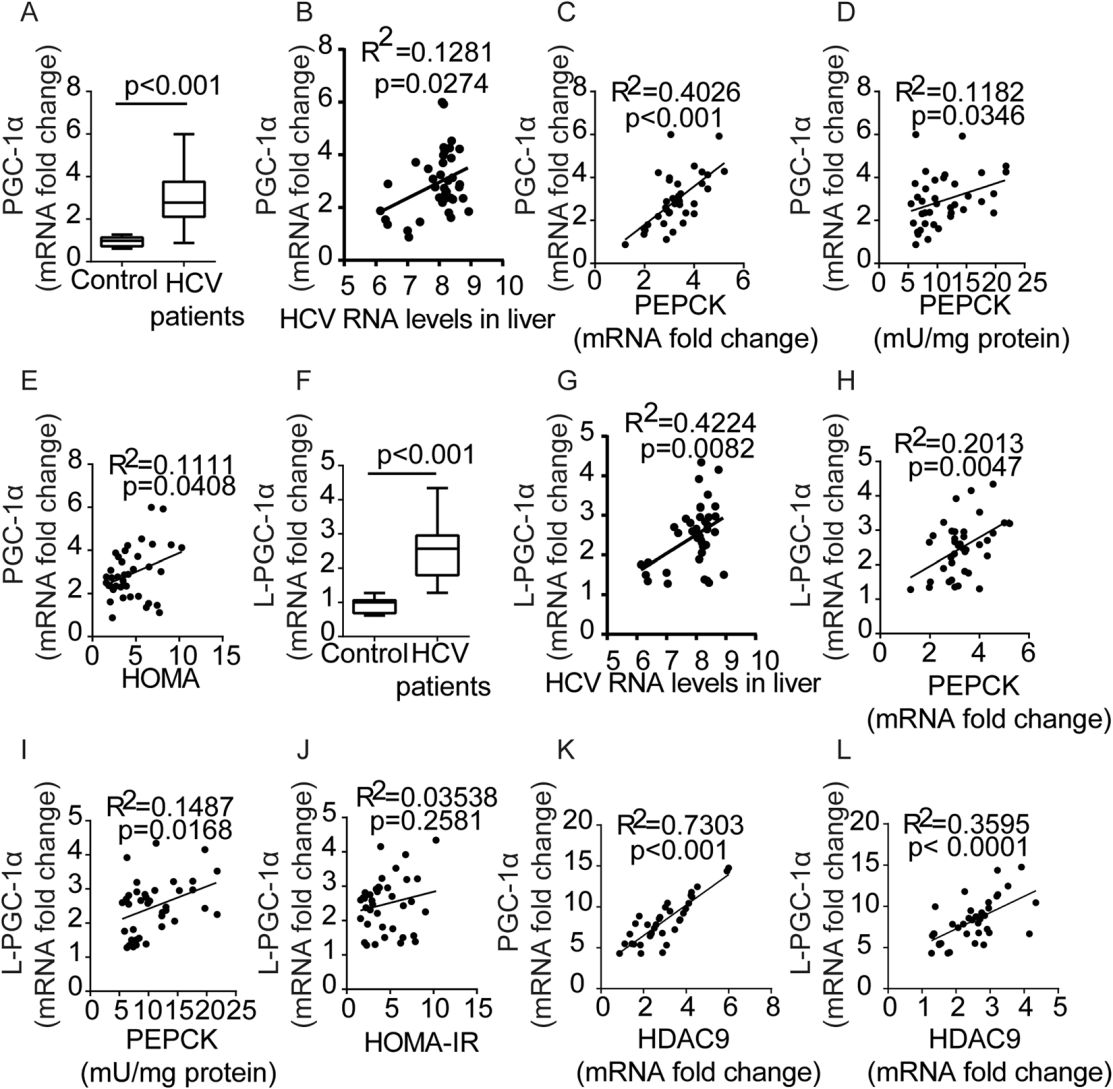
Positive correlation between HDAC9 and PGC-1α in HCV-infected patients. Box plot diagram showing the PGC-1α (**A**) and L-PGC-1α (**F**) mRNA levels in liver biopsies of 15 normal control patients and 38 patients with chronic hepatitis C. Correlation between PGC-1α mRNA levels and PEPCK mRNA levels (**B**), HCV virus load (**C**), PEPCK enzymatic activity (**D**), HOMA-IR score (**E**), or HDAC9 mRNA levels (**K**) in liver. Correlation between L-PGC-1α mRNA levels and PEPCK mRNA levels (**G**), HCV virus load (**H**), PEPCK enzymatic activity (**I**), HOMA-IR score (**J**), or HDAC9 mRNA levels (**L**) in liver. Data are represented as the mean ± SEM for triplicate experiments.

To further evaluate these findings in humans, we examined PGC-1α expression in biopsy samples obtained from the livers of HCV-infected patients. The demographic and clinicopathological characteristics of 38 biopsies obtained from HCV-infected patients and 15 biopsies from normal control patients included in the study are shown in Supplementary Table S2. We observed a statistically significant elevation of PGC-1α expression in liver biopsies taken from HCV-infected patients (Fig. 2A). In the group of 38 HCV-infected patients, PGC-1α mRNA expression level was positively associated with HCV viral load in the liver (Fig. 2B) but not in the serum (data not shown). In HCV-infected subjects, the degree of PGC-1α and PEPCK gene induction appeared to be positively correlated (Fig. 2C). Furthermore, we compared the induction of PGC-1α with PEPCK enzymatic activity and observed a significant positive correlation (Fig. 2D). To determine whether the PGC-1α overexpression observed in patients infected with HCV was correlated with systemic insulin resistance, we measured fasting glucose and insulin levels on the day of the liver biopsy. Indeed, a positive correlation was observed between HOMA-IR values and the induction of PGC-1α in 38 subjects (Fig. 2E).

We also analysed the expression levels of human liver-specific PGC-1α (L-PGC-1α), which results from alternative promoter usage. Similar to PGC-1α, significant elevation of L-PGC-1α expression was observed in HCV patient liver biopsies (Fig. 2F), positively associated with HCV viral loads in the liver (Fig. 2G). A similar positive correlation was found between L-PGC-1α expression levels and PEPCK gene induction or enzymatic activity (Fig. 2H,I). However, the degree of L-PGC-1α induction was not significantly correlated with HOMA-IR values in HCV-infected subjects (Fig. 2J). In a previous study, we reported that HDAC9 regulates hepatic gluconeogenesis via deacetylation of the gluconeogenic transcription factor, FoxO1^13^. Significantly, a strong positive correlation was found between the induction of HDAC9 and PGC-1α (Fig. 2K), as well as L-PGC-1α (Fig. 2L), suggesting that HDAC9 may account for the level of PGC-1α and L-PGC-1α mRNA inductionin HCV-infected hepatocytes.

### HDAC9 strongly activates PGC-1 gene transcription

**Figure 3:**
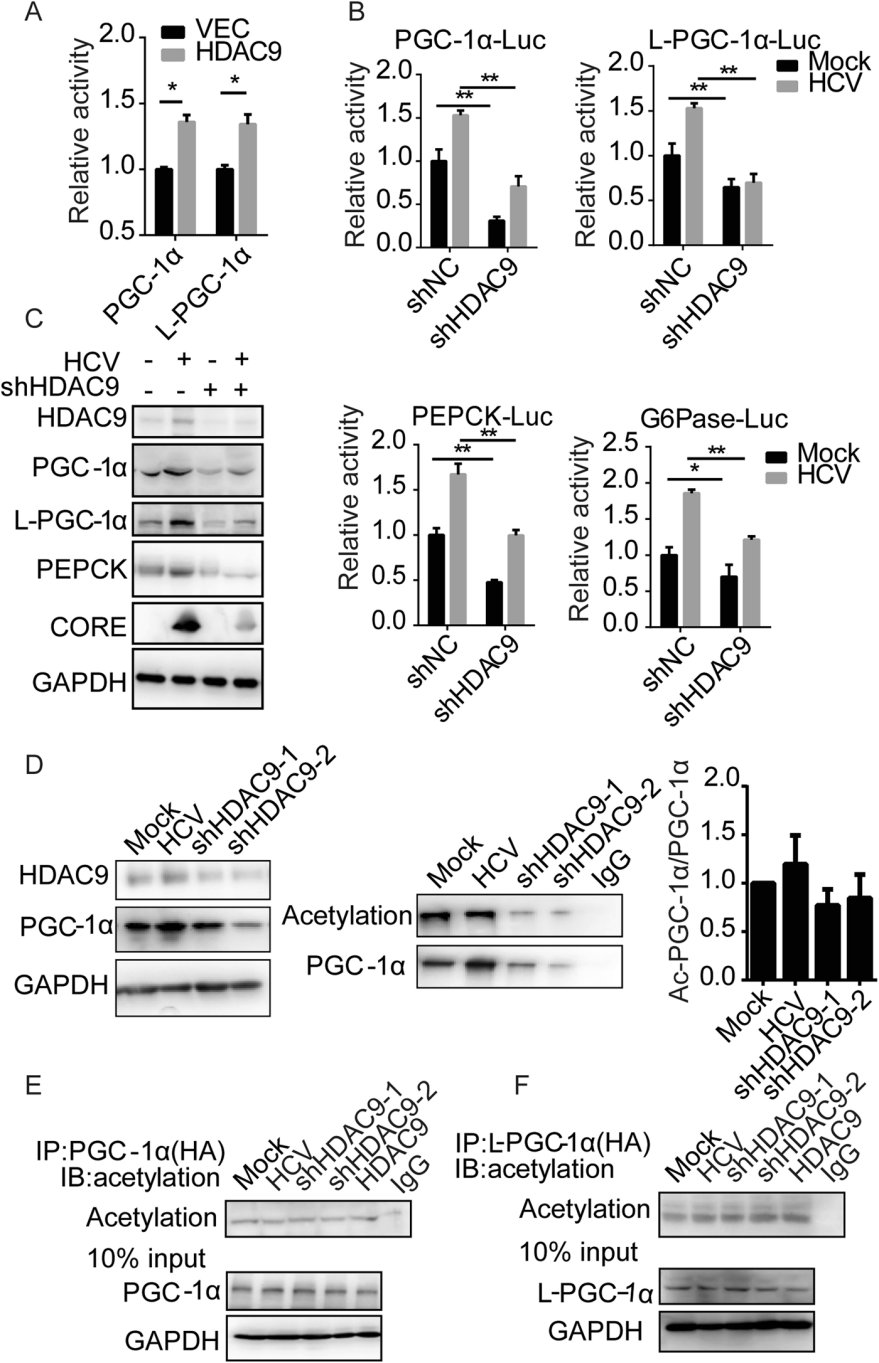
HDAC9 strongly activates PGC-1α gene transcription. (**A**) The promoter activity of PGC-1α and L-PGC-1α in HDAC9-overexpressing cells. (**B**) The promoter activity of PGC-1α, L-PGC-1α (upper panel), PEPCK or G6Pase (lower panel) in HuH7-shHDAC9 cells. Data are represented as the mean ± SEM for triplicate experiments. (**C**) Related protein levels as indicated in (**B**). (**D**) Endogenous PGC-1α (second panel) was immunoprecipitated from the indicated cell lysates and immunoblotted with PGC-1α and acetylated lysine antibodies. The histogram (third panel) represents a densitometric analysis performed to quantify the relative intensity of Ac-PGC-1α/PGC-1α-immunoreactive bands detected by Western blotting. Exogenous PGC-1α (**E**) and L-PGC1α (**F**) were immunoprecipitated by HA antibody from the indicated cell lysates and immunoblotted with acetylated lysine antibodies. The whole cell lysate for immunoprecipitation was immunoblotted with the antibodies indicated in (**D**-**F**). Goat-anti-rabbit-IgG or Rabbit-anti-mouse was used as the negative control in (**D**–**F**).

We next examined whether HDAC9 was involved in activation of the PGC-1α or L-PGC-1α promoter. As shown in Fig. 3A, transfection of an HDAC9 expression vector markedly induced the activity of both the PGC-1α and L-PGC-1α promoters in hepatocytes. Furthermore, HDAC9 silencing significantly attenuated the activity of the PGC-1α and L-PGC-1α promoters, in parallel with the decreased activity of PEPCK and G6Pase promoter in both HuH7 cells and HCV-infected cells (Fig. 3B,C). Data shown in Fig. 3C indicate that activation of the PGC-1α promoter led to increased expression of PGC-1α and its target gene PEPCK. Consistently, glucose production in HuH7 cells and HCV-infected cells was dramatically reduced compared to the control following HDAC9 knockdown, as reported previously (Supplementary Fig. S2).

We examined whether HDAC9 influences the acetylation of PGC-1α. Downregulation of PGC-1α proteins was observed following HDAC9 depletion (Fig. 3D). However, endogenous PGC-1α acetylation was comparable in HCV-infected cells and in HuH7-shHDAC9 cells (Fig. 3D). Furthermore, we transfected PGC-1α and L-PGC-1α expression vectors with HA-tag, respectively. As shown in Fig. 3E, neither HDAC9 depletion nor HDAC9 overexpression changed the status of PGC-1α acetylation, similar to the effects observed for L-PGC-1α (Fig. 3F).

Thus, in contrast to FoxO1, HDAC9 is not involved in modulating the acetylation of PGC-1α but rather the expression of PGC-1α.

### HDAC9 is involved in inducing PGC-1α promoter activity by deacetylation of FoxO1 and upregulation of CREB

**Figure 4:**
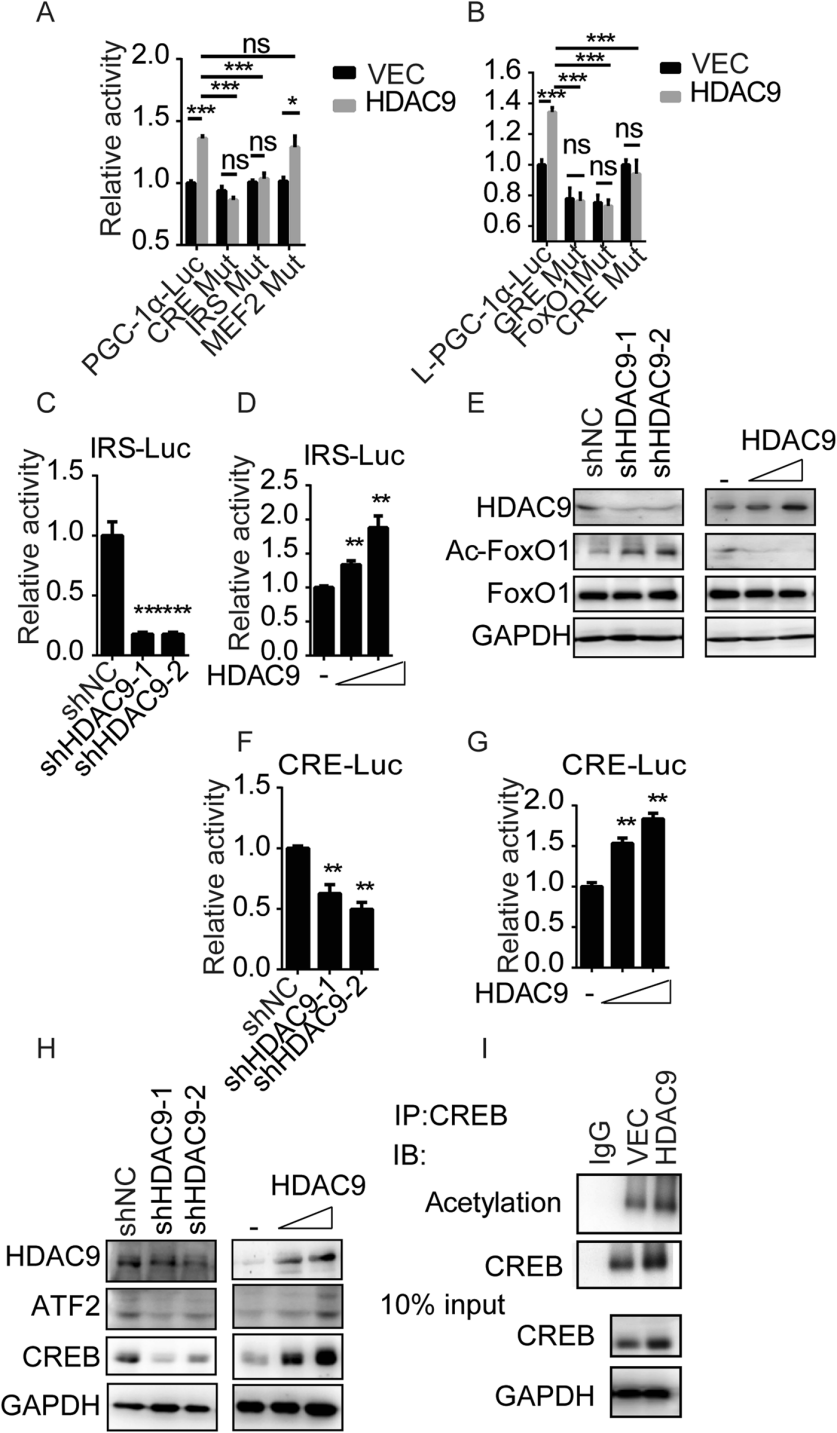
Deacetylation of FoxO1 and upregulation of CREB by HDAC9-induced PGC-1α promoter activity. Different PGC-1α (**A**) and L-PGC-1α (**B**) promoter mutant activities in HDAC9-overexpressing cells, as detected using luciferase reporter assay. IRE-Luc activity was detected following HDAC9 depletion (**C**) or by dose response upon HDAC9 induction (**D**). (**E**) The proteins indicated in (**C**) or (**D**) were analysed. CRE-Luc activity induced by HDAC9 depletion (**F**) or dose response following HDAC9 induction (**G**). (**H**) The proteins indicated in (**F**) or (**G**) were analysed. (**I**) Endogenous CREB (right panel) was immunoprecipitated from the indicated cell lysates and immunoblotted with antibodies against CREB and acetylated lysine. Data are represented as the mean ± SEM for triplicate experiments.

Because the HDAC9 protein lacks a DNA binding domain, the PGC-1α promoter is obviously not a direct target for HDAC9. Several transcription factors, including myocyte enhancer factor 2 (MEF2), FoxO1, and CREB, have been shown to regulate PGC-1α expression in response to physiologic stimuli^19^. To determine the effect of these transcription factors on PGC-1α elevation following HDAC9 overexpression, we performed reporter assays using IRS-, CRE-, and MEF2-mutant promoter constructs (Supplementary Fig. S3)^9^. Compared with intact PGC-1α promoter, the deletion of the IRS or CRE motif reduced HDAC9-mediated activation by almost 50%, whereas no inhibitory effect was observed using the MEF2-deleted promoter constructs (Fig. 4A). Together, these findings demonstrate that both the IRS and CRE-binding sites are necessary for the transactivation of PGC-1α by HDAC9.

The expression of L-PGC-1 is regulated by CREB, FoxO1 and glucocorticoid signalling^9^. We therefore analysed the effect of mutations in the L-PGC-1α promoter region (Supplementary Fig. S3). HDAC9 overexpression significantly increased L-PGC-1α promoter activity, whereas no effect was observed on CRE-, GRE-, or FoxO1-deleted constructs from L-PGC-1α promoter region (Fig. 4B). This result suggests that the HDAC9-induced upregulation of L-PGC-1α is dependent on the FoxO1, CRE, or GRE motif.

We next tested the direct effect of HDAC9 on the IRS- and CRE-responsive luciferase construct. As shown in Fig. 4C, the depletion of HDAC9 depressed the activities of IRS-Luc and elevated FoxO1 acetylation in parallel (Fig. 4E), whereas HDAC9 overexpression stimulated IRS-Luc activity (Fig. 4D) and decreased the acetylation of FoxO1 (Fig. 4E). These results are consistent with data from a previous study in which we showed that the upregulation of HDAC9 enhanced FoxO1 transcript activity via FoxO1 deacetylation in hepatocytes^13^.

In a parallel experiment, CRE-Luc was detected following the knockdown and overexpression of HDAC9, respectively. This activity was susceptible to inhibition by HDAC9 depletion (Fig. 4F), whereas HDAC9 overexpression stimulated activity (Fig. 4G). Data shown in Fig. 4I reveal that CREB acetylation did not change dramatically following HDAC9 expression. Interestingly, the expression of CREB was positively correlated with the overexpression of HDAC9, while CREB protein levels were reduced by knockdown of HDAC9 (Fig. 4H).

Together, these results indicate that HDAC9-induced deacetylation of FoxO1 and upregulation of CREB increased PGC-1α promoter activity.

### The HDAC9-FoxO1 signalling axis is required for the regulation of gluconeogenic transcription factors

**Figure 5:**
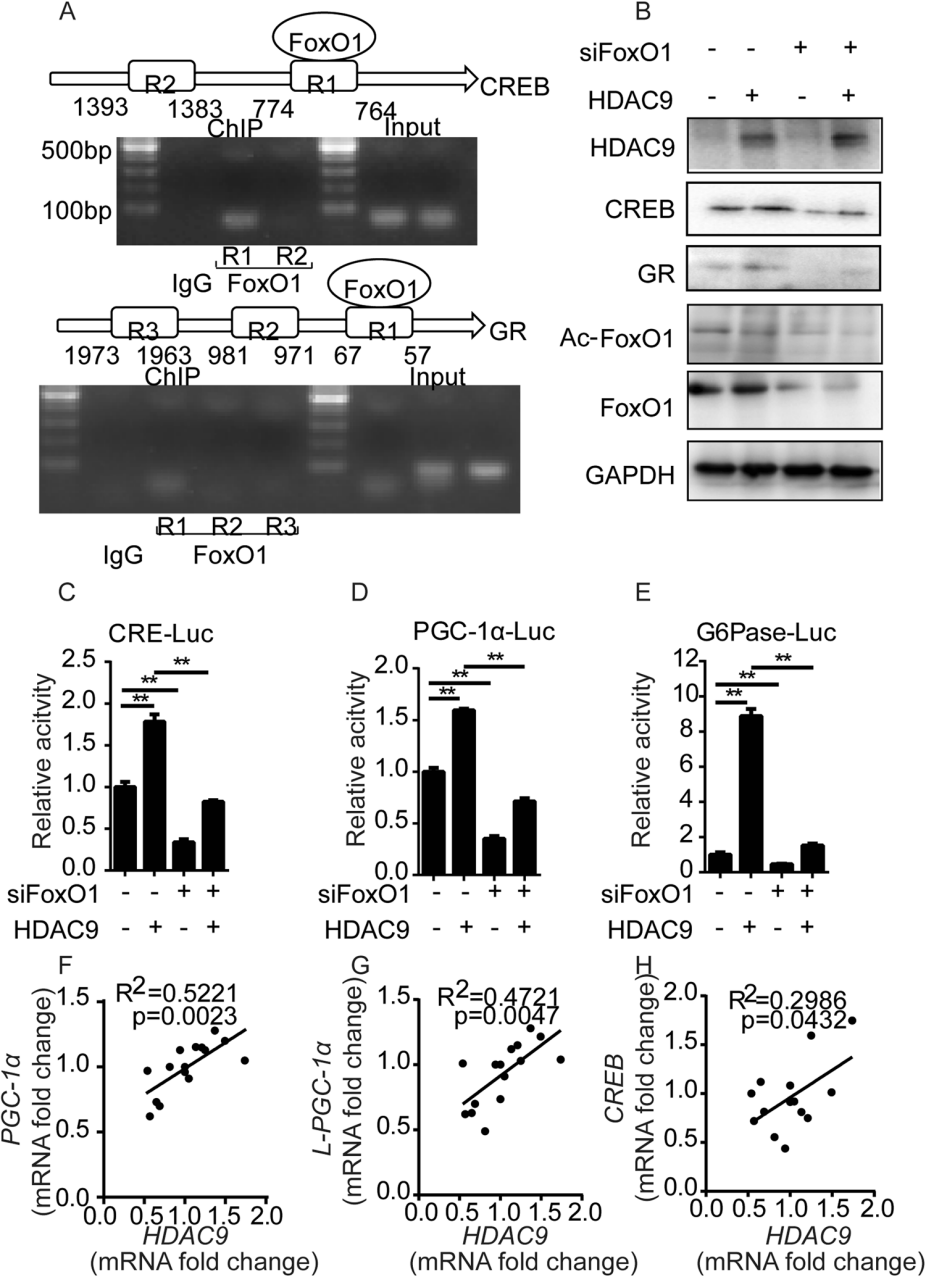
HDAC9-FoxO1 signalling is required for the regulation of gluconeogenic transcription factors. (**A**) *In vivo* binding of FoxO1 to chromatin sites in CREB and GR. The positions of FoxO1 binding sites are indicated within the CREB and GR proximal promoter regions. Chromatin immunoprecipitation assays with an antibody to FoxO1 were used to detect FoxO1 bound to the proximal CREB or GR promoter in HuH7 cells. Rabbit IgG was used as a negative control. (**B**) The indicated proteins were analysed following HDAC9 overexpression, FoxO1 knockdown, or a combination of the two. (**C**–**E**) CRE-Luc (**C**), PGC-1α promoter (**D**), and G6Pase promoter (**E**) activities as in (**B**). (**F**–**H**) A positive correlation between HDAC9 mRNA levels and PGC-1α (**F**), L-PGC-1α (**G**), or CREB (**H**) mRNA level was observed in normal individuals. Data are represented as the mean ± SEM for triplicate experiments.

We next examined the mechanism by which HDAC9 regulates the expression of CREB. *In silico* analysis predicted two FoxO1 binding sites in the putative promoter region of the CREB locus. Chromatin immunoprecipitation analysis revealed that the CREB gene was indeed a target of FoxO1 (Fig. 5A). We proceeded to investigate whether FoxO1 is involved in regulating the expression of CREB. Indeed, knockdown of FoxO1 markedly reduced the expression of CREB (Fig. 5B), similar to the effect of HDAC9 depletion (Supplementary Fig. S4A). Meanwhile, CRE-Luc activity was also reduced by knocking down FoxO1 (Fig. 5C, Supplementary Fig. S4B). We further investigated whether HDAC9-FoxO1 signalling is involved in regulating the expression of CREB. Transfection of HuH7 cells with an HDAC9 expression vector induced significant CREB expression, whereas the upregulation of CREB by HDAC9 decreased following FoxO1 knockdown (Fig. 5B). These results indicate that HDAC9-induced deacetylation of FoxO1 upregulates CREB expression. Moreover, CRE-Luc activity exhibited a pattern similar to that of CREB expression (Fig. 5C). FoxO1 depletion attenuated the HDAC9-induced promoter activity of both PGC-1α (Fig. 5D) and G6Pase (Fig. 5E), further suggesting the participation of HDAC9-FoxO1 signalling in the regulation of gluconeogenic transcription factors and gluconeogenic enzymes.

We further confirmed the relationship betweenHDAC9 and PGC-1α or CREB in normal individuals. A strong positive correlation was observed between HDAC9 mRNA abundance and PGC-1α or L-PGC-1α (Fig. 5F–G) in normal individuals. Moreover, a positive correlation was also found between the levels of CREB and HDAC9 mRNA (Fig. 5H).

Together, HDAC9-induced deacetylation of FoxO1 contributed to the upregulation of both PGC-1α and CREB; furthermore, the HDAC9-FoxO1 signalling axis may participate in the regulation of gluconeogenic-related transcription factors and gluconeogenesis.

## Discussion

HDACs regulate the acetylation of histones and transcriptional factors involved in glucose homeostasis and play a central role in the regulation of glucose metabolism^11^. In this study, we demonstrated that HDAC9, a class IIa HDAC, is involved in modulating gluconeogenesis in the liver by regulating the expression of gluconeogenic transcription factors, such as PGC-1α and CREB, via deacetylation of the FoxO1 transcription factor. In particular, the HDAC9-FoxO1 signalling axis is strongly induced upon HCV infection, which promotes the expression of gluconeogenic genes such as PEPCK and gluconeogenic transcription factors to enhance gluconeogenesis. These results suggest a gene regulatory network involved in HCV-induced abnormal glucose homeostasis and T2DM.

Recent studies have shown that class IIa HDACs, including HDAC 4, 5, and 7, regulate FoxO and glucose homeostasis responses to the fasting hormone glucagon in the liver^12^. Our previous studies have indicated that nuclear HDAC9 also plays a role in regulating gluconeogenesis and glucose homeostasis via deacetylating nuclear FoxO1. We found that HDAC9 also regulates PGC-1α, thereby enhancing its association with gluconeogenic gene promoters, leading to acute transcriptional induction of gluconeogenic enzymes. Unlike FoxO1 proteins, which are regulated by post-translational modification responses to HDAC9 in the liver, HDAC9 regulates PGC-1α specifically by eliciting changes in gene expression levels. HDAC9 suppression in hepatocytes inhibits the expression of PGC-1α and other gluconeogenic genes. Notably, a positive association was identified between PGC-1α and HDAC9 gene expression in normal individuals, consistent with a recent report of positive correlation between PGC-1α mRNA and HDAC9 mRNA levels immediately post-exercise in skeletal muscle^20^. Thus, our findings suggest that HDAC9, like PGC-1α, could induce the expression of CREB, another gluconeogenic transcription factor, to enhance its transactivation potential.

Phosphorylation modulates CREB activity by altering its subcellular localization or DNA binding ability^6^. In our study, we characterized both transcriptional and post-translational regulation of CREB. CREB is important not only in the direct transcriptional activation of gluconeogenic genes but also in modulating the fasting-mediated transcriptional activation of PGC-1α^4, 7^. Herein, we found that HDAC9-upregulated CREB stimulates the gluconeogenic programme directly by binding to the PEPCK and G6Pase gene promoters and to PGC-1α. In addition, we found that GR, which serves as a crucial transcriptional regulator that orchestrates the activation of gluconeogenic genes^5^, was also induced by HDAC9 in the liver (Fig. 5B, Supplementary Fig. S5A).Thus, HDAC9 may play a role in regulating the expression of gluconeogenic transcription factors or key enzymes and gluconeogenesis in hepatocytes.

FoxO1 regulates multiple metabolic pathways in the liver, including gluconeogenesis, glycolysis, and lipogenesis^3^. HDAC9 may cause the deacetylation of nuclear FoxO1, thereby enhancing FoxO1 DNA-binding activity and its association with gluconeogenic gene promoters. Our findings reveal that FoxO1 knockdown decreased the activity of the HDAC9-induced PGC-1α promoter. Because PGC-1α is regulated by FoxO1 at the transcriptional level^8^, HDAC9-activated FoxO1 stimulated the gluconeogenic programme directly by binding to the PEPCK and G6Pase promoters. In parallel, it increased the expression of gluconeogenic genes via a feedforward mechanism involving the induction of PGC-1α. In addition, FoxO1 depletion attenuatedHDAC9-induced CRE-Luc and CREB expression, further demonstrating a role for FoxO1 in HDAC9-induced regulation of gluconeogenic transcription factors. Chromatin immunoprecipitation analysis revealed that both the CREB and GR genes are targets of FoxO1 (Fig. 5A), suggesting that the HDAC9-FoxO1 signalling axis could play an important role in modulating the expression of gluconeogenic transcription factors such as CREB, GR and PGC-1α.In addition, transcriptional co-activators for CREB or FoxO1 [such as CREB regulated transcription co-activator 2 (CRTC2), protein arginine methyltransferases (PRMTs)] and transcriptional repressors for CREB or FoxO1 (such as SHP, also known as NR0B2) may be involved in this pathway^6, 21–24^. Analyses integrating HDAC9-FoxO1 signalling and each transcriptional regulator will be necessary to delineate the precise transcriptional regulatory mechanisms involved in hepatic gluconeogenesis.

Recent research has resulted in the view that virus-induced changes to a host’s metabolism can be detrimental to its life cycle^25^. Here, we report that the metabolite pattern in hepatocytes following HCV infection could shift to exaggerate gluconeogenesis. Our clinical study showed that HDAC9 expression levels and gluconeogenic activity were elevated in liver tissues obtained from HCV-infected patients. Upregulation of both HDAC9 and PGC-1α expression and the positive correlation between the induction of HDAC9 and PGC-1α further confirmed the regulation of PGC-1α expression by HDAC9. To our surprise, HCV infection did not result in significant upregulation of CREB expression, whereas CREB5 (also known as CRE-BPA)^26^, another CRE binding protein, was dramatically elevated, suggesting a key role for CREB5 following HCV infection (Supplementary Fig. S4C). Moreover, HDAC9 overexpression markedly induced CRE-Luc activity and elevated the level of CREB5 mRNA, which is reduced by FoxO1 knockdown (Supplementary Fig. S4D). The presence of two predicted FoxO1 binding sites in the putative promoter of CREB5 further suggests the involvement of the HDAC9-FoxO1 signalling axis in CREB5 regulation. It is therefore of interest to investigate the mechanisms by which CREB5 or CREB responds to stimuli to regulate glucose homeostasis. Thus, HCV infection may lead to abnormal glucose homeostasis via HDAC9 upregulation, resulting in the enhancement of FoxO1 DNA-binding activity and its association with the promoters of gluconeogenic transcription factors and rate-controlling gluconeogenic enzymes, finally increasing hepatic gluconeogenesis. This proposed mechanism suggests the HDAC9-FoxO1 signalling axis as a potential therapeutic target for suppressing hepatic gluconeogenesis and lowering blood glucose level. HDAC inhibitors (HDACis) targeting class I, II, and IV HDACs are currently under development for use as anticancer agents following FDA approval such as Vorinostat and Romidepsin^27^. The utility of HDAC inhibitors for treating metabolic disease therefore merits consideration.

In conclusion, the present study shows the key role of the HDAC9-FoxO1 signalling axis in regulating gluconeogenic genes, transcriptional factors, gluconeogenesis metabolism, and HCV-induced gluconeogenesis in hepatocytes. Our findings provide novel insights into the roles of key metabolic regulators in hepatic cells and clues to possible mechanisms underlying the development of HCV-induced glucose abnormality.

## Methods

### Patients and biopsies

All patients, their relatives, or both provided written informed consent for use of their clinical and pathological information for research purposes as well as its storage in the hospital’s database. The Ethics Committee of the First Hospital of Jilin University approved the methods and experimental protocols used in the present study, which were performed in accordance with the ethical standards of our institutional research committee and the tenets of the 1964 Declaration of Helsinki and its amendments, or comparable ethical standards. Methods were performed in accordance with the approved guidelines. All human tissue samples were collected from the Liver Unit of the First Hospital of Jilin University. Human liver tissue samples were obtained via fine needle biopsy from 38 HCV-infected patients. Normal human liver tissue was obtained from either spare donor tissue intended for transplantation or normal liver tissue resected from patients with benign hepatic tumours. Diagnosis of chronic HCV infection in patients and analysis of all biopsies were based on standard serological assays and the presence of abnormal serum aminotransferase levels for at least six months. All HCV patients tested positive for HCV antibody based on a third-generation ELISA test. HCV infection was confirmed by detection of circulating HCV RNA using an HCV PCR-based assay (Qiagen). At time of biopsy, liver tissue (2–3 mm) was immediately frozen in TRIzol and stored at −80 °C. Fasting glucose and insulin levels were measured on the days of biopsy. Insulin resistance was assessed by the HOMA-IR score [homeostasis model assessment, calculated as (fasting insulin × fasting glucose)/22.5]. FLEXMAP 3D quantification (Luminex, TX) of concentrations of insulin and C-peptide were also performed.

### Cells and virus

Human hepatoma HuH7 cells (kindly provided by Frank Chisari) were cultured as previously described^28^. The HCV J399EM strain was derived using JFH-1 virus by insertion of enhanced green fluorescent protein into the HCV NS5A region^29^. Virus production and infection was performed as described previously^28^. Mock-infected controls were generated in parallel to virus infections. For infection *in vitro*, HuH7 cells (1 × 10^6^) were infected with J399EM for the indicated times with the indicated multiplicities of infection (MOI).

### Metabolite Profiling

Extraction of intracellular metabolites and nuclear magnetic resonance (NMR) analysis were performed as described^30^.

### Plasmids, transfection and luciferase reporter assays

PEPCK-Luc and G6Pase-Luc (kindly provided by Prof. Akiyoshi Fukamizu) were constructed by cloning 671-bp and 834-bp upstream fragments into the promoter-luciferase reporter vector pGL3-basic^8^. PGC-1α-Luc and L-PGC-1α-Luc constructs were generated by cloning 3.518-kb and 2.614-kb upstream fragments, respectively, into pGL4.17. Mutants of the PGC-1α promoter region [IRS-Mut (which was mutated in all three insulin response sequences), CRE-Mut, MEF2-Mut] and mutants of the L-PGC-1α promoter region [glucocorticoid response element (GRE)-Mut, FoxO1-Mut, and CRE-Mut] were created by using site-directed mutagenesis (Supplementary Fig. S3) (kindly provided by Prof. Tao Peng)^9^. The constructs expressing CRE-Luc were purchased from Stratagene. pRL-SV40-Renilla reporter plasmid (Promega) served as a transfection control. Luciferase reporter assays were performed as described^18^. Plasmids expressing HDAC9 were generated from the pXJ40-HA vector with the indicated primers (Supplementary Table S3) and were transfected into HuH7 cells (at 1 μg or 5 μg) using Lipofectamine 2000 (Invitrogen).

### Real-time PCR and Western blotting

RNA isolation, cDNA synthesis, and quantitative PCR with the indicated primers (Supplementary Table S3) as well as Western blotting were performed as described^28^. The following antibodies were used: NS3, ab13830, CORE, ab2740, HDAC9, and ab18970 (Abcam); FoxO1, 9454, PEPCK, 12940, GAPDH, 2118, ATF2, 9226, Acetylated-Lysine, 9681, PGC-1α, and 2178 (Cell Signalling Technology); and Acetyl-FoxO1, sc49437, PGC-1α, and sc-5815 (Santa Cruz Biotechnology).

### Stable cell line construction

The two sequences (Supplementary Table S3) encoding short hairpin RNA (shRNA) targeting the HDAC9 gene (shHDAC9-1 and shHDAC9-2) and a negative-control shRNA were cloned into the shRNA expression vector pSUPER.retro.neo (OligoEngine, Inc.) following the manufacturer’s instructions. Stable cell lines named HuH7-shNC (shNC) and HuH7-shHDAC9 (shHDAC9-1, also indicated as shHDAC9; shHDAC9-2) were generated as described^31^.

### RNA interference

For knockdown experiments, 50 pmol small interfering RNA (siRNA) specific for HDAC9 (Qiagen, GS9734), PGC-1α (Qiagen, GS10891), and FoxO1 (GGAGGUAUGAGUCAGUAUATT)^32^ and a negative control (Qiagen, 1027310) were transfected into HuH7 cells via Lipofectamine 2000.

### Glucose production assay

The production of glucose was measured using an Amplex® Red Glucose/Glucose oxidase assay kit (Invitrogen) according to the manufacturer’s instructions^33^.

### Co-immunoprecipitation and Chromatin Immunoprecipitation (ChIP)

Co-immunoprecipitation assays were performed as described^28^. ChIP analysis was performed following the procedures described by Daftari *et al*. with the appropriate primers (Supplementary Table S3)^34^.

### PEPCK, G6Pase, FBPase activity assay

PEPCK enzyme activity was assayed using an NADH-coupled system^35^. G6Pase activity was assessed for portions of intact microsomes as described^36^. FBPase activity was measured spectrophotometrically according to the method described by Charles^37^.

### Statistical analysis

Data are presented as the means ± SEM. Statistical analysis was carried out using Student’s t-test when comparing two groups and ANOVA when comparing multiple groups. Differences were considered significant at P < 0.05 (*p < 0.05; **p < 0.01; ***p < 0.001). Statistical analyses were performed using GraphPad Prism.

### Ethics Statement

All patients and/or their relatives provided written informed consent for their clinical and pathological information to be used for research and to be stored in the hospital database; this study, including its methods and experimental protocols, was approved by the Ethical Committee of the First Hospital of Jilin University. All procedures performed in our study were done so in accordance with the ethical standards of our institutional research committee and the 1964 Helsinki Declaration and its later amendments or comparable ethical standards. Methods were carried out in accordance with the approved guidelines.

## Electronic supplementary material

Supplementary data
