# Author Correction: Role of HDAC9-FoxO1 Axis in the Transcriptional Program Associated with Hepatic Gluconeogenesis

**DOI:** 10.1038/s41598-020-66085-8

**Published:** 2020-05-28

**Authors:** Jizheng Chen, Zhilei Zhang, Ning Wang, Min Guo, Xiumei Chi, Yu Pan, Jing Jiang, Junqi Niu, Sulaiman Ksimu, John Zhong Li, Xinwen Chen, Qian Wang

**Affiliations:** 1 Jiangsu Province Key Lab of Human Functional Genomics, Department of Biochemistry and Molecular Biology, Nanjing Medical University, Nanjing, 210029 China; 2 State Key Lab of Virology, Wuhan Institute of Virology, Chinese Academy of Sciences, Wuhan, 430071 China; 3 State Key Laboratory of Natural Medicines, School of Life Science and Technology, China Pharmaceutical University, Nanjing, Jiangsu 210009 China; 4 Department of Hepatology, The First Hospital of Jilin University, Changchun, 130021 China; 5 The Center for Technology and Education, The first Affiliated Hospital of Xinjiang Medical University, Urumchi, 830054 China

Correction to: *Scientific Reports* 10.1038/s41598-017-06328-3, published online 21 July 2017

This Article contains errors in the Results section and the Supplementary Data.

In the Results, under the subheading ‘HCV infection redirects cellular metabolism towards gluconeogenesis’,

“Consistent with this pattern, HCV-infected cells showed lower levels of intracellular glucose (Fig. 1B) and produced more lactate (Fig. 1C)”

should read:

“Consistent with this pattern, HCV-infected cells showed higher levels of intracellular glucose (Fig. 1B) and produced more lactate (Fig. 1C)”.

In Supplementary Table S3, the primers for HDAC9 are missing, and the shHADC9 sequences displayed are incomplete. The correct Supplementary Table S3 appears below as Table 1.

**Table 1:** Primers for qPCR and ChIp and sequences of shRNA used in this study.

Strand/Gene	Primer	Sequence(5’-3’)
*PGC-1α*	PGC1F	TGAAAAAGCTTGACTGGCG
	PGC1R	AAGATCTGGGCAAAGAGGC
*L-PGC-1α*	LPGC1F	TCCCTCTGTTGCCTTTGTG
	LPGC1R	TAACCCCATGCCATCCAT
*CREB*	CREBF	ATTCACAGGAGTCAGTGGATAGT
	CREBR	CACCGTTACAGTGGTGATGG
*CREB5*	CREB5F	AAAGACTGCCCAATAACAGCC
	CREB5R	AAGCTGGGACAGGACTAGCA
*HCV*	HCVF	CCCTATCAGGCAGTACCACAAGG
	HCVR	ACCTGCCCCTAATAGGGGCG
*Actin*	ActinF	GAAATCGTGCGTGACATTAA
	ActinR	AAGGAAGGCTGGAAGAGTG
*CREB-CHIP-1*	CREBCF1	CCCCGCCTTTCTCCAGACAACA
	CREBCR1	GTAGAAAACAGGCTGAGGAAAG
*CREB-CHIP2*	CREBCF2	TCTTTAGGAAGCAGAGAGGGTT
	CREBCR2	GGAAAGGTTGAGAGTAACAGAA
*GR-CHIP-1*	GRCF1	ATGTGACTCCAACATGCTTTTG
	GRCR1	CAAAAGCATGTTGGAGTCACAT
*GR-CHIP-2*	GRCF2	TGTCAAAGAGCATGTCTCTGGC
	GRCR2	GAATTATCATTGTGCGTGGCAC
*GR-CHIP-3*	GRCF3	CTTATCTACGGAGGATATGTTC
	GRCR3	TGAGTTATTGTTAATCTCTTGC
*HDAC9*	HDAC9F	CGCGAATCCATGCACAGTATGATCAGCTC
	HDAC9R	CGCCTCGAGTCACAAGGCTGGCTCCTCTTC
**Oligo name**	**Sequence**	
shHDAC9-1	CCGGCAAACTGCTTTCGAAATCTATCTCGAGATAGATTTCGAAAGCAGTTTGTTTTTTG	
shHDAC9-2	CCGGGAGCAGTTAATAGGCTTTAAACTCGAGTTTAAAGCCTATTAACTGCTCTTTTTTG	

