# Supplementary material for: Role of HDAC9-FoxO1 Axis in the Transcriptional Program Associated with Hepatic Gluconeogenesis
Source: Sci Rep. 2017 Jul 21;7:6102. doi: 10.1038/s41598-017-06328-3 (PMC5522426; doi:10.1038/s41598-017-06328-3)
Supplement: Supplementary file 1 — Supplementary data [file 41598_2017_6328_MOESM1_ESM.pdf]

Role of HDAC9-FoxO1 Axis in the Transcriptional Program Associated with Hepatic Gluconeogenesis

Jizheng CHEN[2], Zhilei ZHANG[1], Ning WANG[1], Min GUO[3], Xiumei CHI[4], Yu PAN[4], Jing JIANG[4], Junqi NIU[4], Sulaiman Ksimu[5], Zhong LI[1], Xinwen Chen[2], and Qian WANG[1*]

Supplementary Figure S1

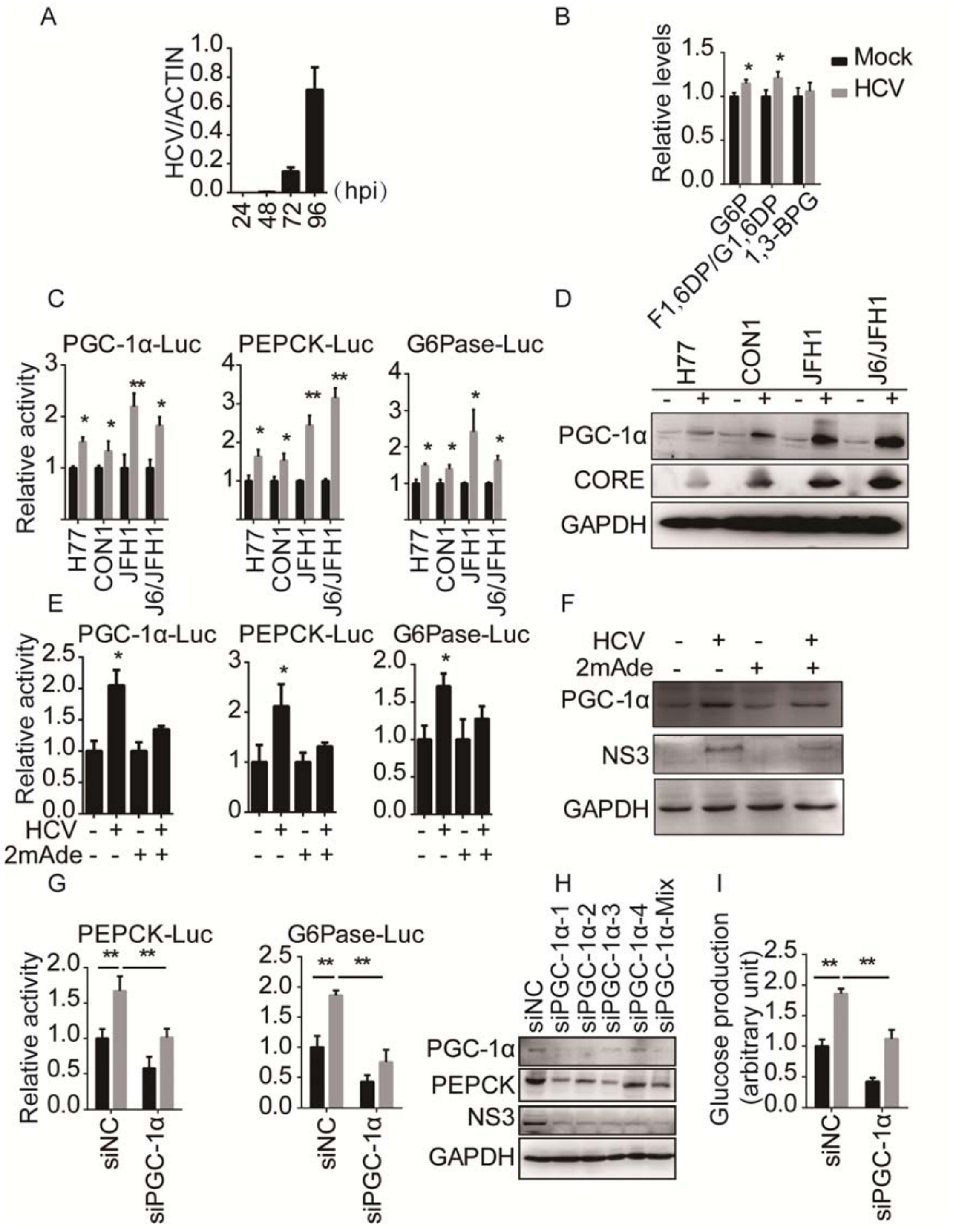

Supplementary Figure S1. HCV infection redirects cellular metabolism towards gluconeogenesis.

(A) HCV RNA levels in mock- or HCV-infected-HuH7 cells (1.0 MOI) at indicated time points (hours post infection, hpi). (B) Levels of glycolytic intermediates at 24 hpi.

The promoter activity of PGC-1α, PEPCK, G6Pase (C), and related protein (D) levels in H77, CON1 cells, compared with HuH7 control cells. The promoter activity of PGC-1α, PEPCK, G6Pase (E), and related protein (F) levels in mock- or HCV-infected (1.0 MOI)-cells treated with 2mAde (10 mM, 96 h). The promoter activity of PEPCK and G6Pase (G), and related protein (H) levels in mock- or HCV-infected (1.0 MOI)-cells pretransfected with PGC-1α siRNAs for 48 h at 96 hpi. (I) Relative cellular glucose production as in (G). Data are represented as mean ± SEM. # indicates undetectable levels. *p<0.05; **p<0.001.

Supplementary Figure S2

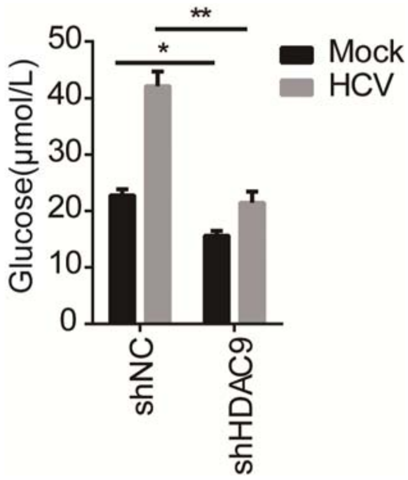

Supplementary Figure S2. HDAC9 Strongly Activate PGC-1α Gene Transcription.

Relative cellular glucose production in HDAC9 shRNA cells. Data are represented as mean ± SEM for triplicate experiments.

Supplementary Figure S3

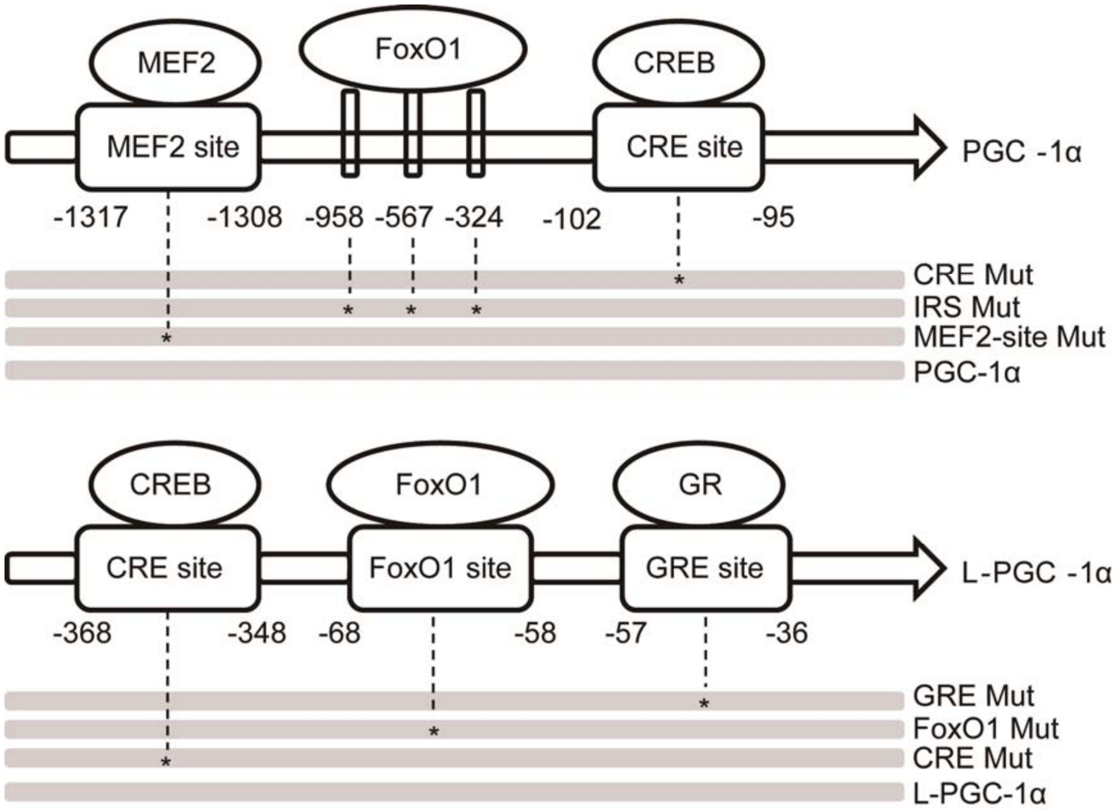

Supplementary Figure S3. Schematic of the PGC-1α and L- PGC-1α promoter site-specific mutants.

Schematic of the three PGC-1α promoter site-specific mutants. IRS, insulin response sequence; CRE, cAMP response element; MEF2, myocyte enhancer factor 2. Schematic of the three L-PGC-1α promoter site-specific mutants. CRE, cAMP response element; GRE, glucocorticoid response element; FoxO1 site.

Supplementary Figure S4

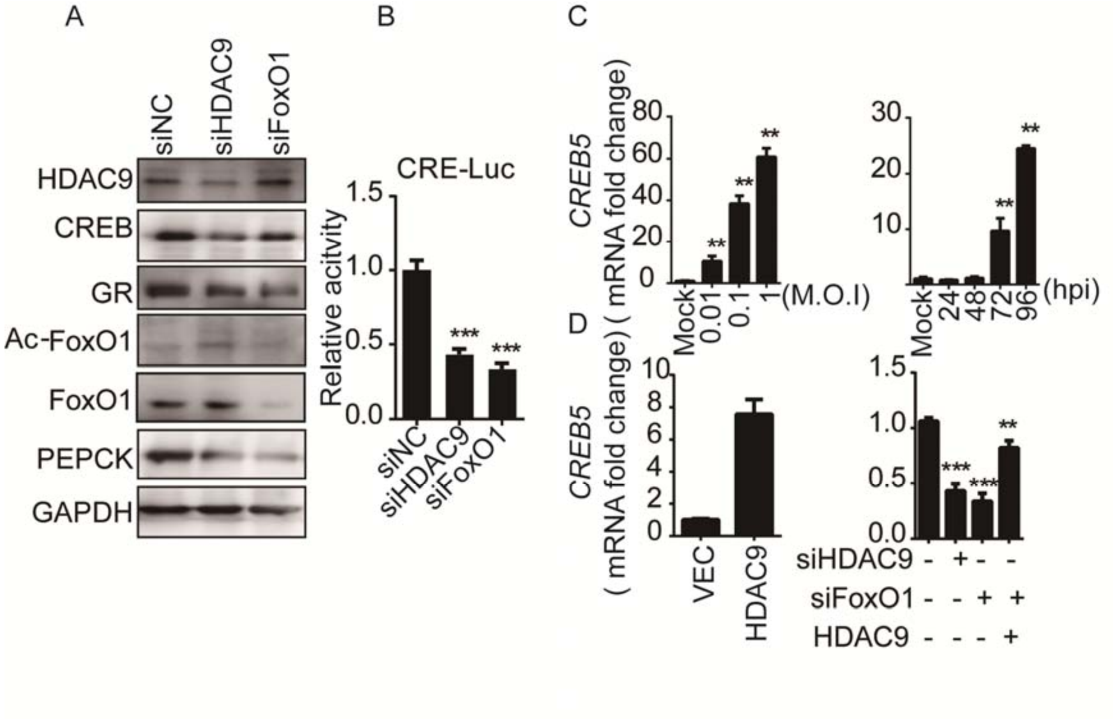

Supplementary Figure S4. HDAC9-FoxO1 signaling axis is required for the regulation of gluconeogenic transcription factors.

Indicated proteins (A) and CRE-Luc (B) were analyzed followed by HDAC9 or FoxO1 silence. (C) CREB5 mRNA levels in mock- or HCV-infected-HuH7 cells (1.0 MOI) at indicated time points (Hours post infection, hpi). (D) CREB5 mRNA levels were analyzed followed by HDAC9 overexpression, FoxO1 knockdown, or a combination of the two. Data are represented as mean ± SEM for triplicate experiments.

Supplementary Table S1. Metabolites analysis in this study.

BIOCHEMICAL NAME	24 hr	48 hr	72 hr	96 hr	24 hr Mean Values		48 hr Mean Values		72 hr Mean Values		96 hr Mean Values	
	Fold Change	Fold Change	Fold Change	Fold Change	HCV+	HCV -	HCV+	HCV -	HCV+	HCV -	HCV+	HCV -
glucose	0.7389	0.8326	1.2785	1.9554	6.4480	8.7267	0.9838	1.6059	0.8199	0.6413	0.6542	0.3346
glucose-6-phosphate (G6P)	1.1503	1.2173	1.3895	1.5726	1.8088	1.5724	1.4093	1.1577	1.5292	1.1005	1.6790	1.0677
Isobar: fructose 1,6-diphosphate, glucose 1,6-diphosphate (F1,6DP/G1,6DP)	1.2106	1.2155	2.3598	4.5166	2.8818	2.3805	2.1325	1.7544	2.0158	0.8542	1.9987	0.4425
1,3-diphosphoglycerate (1,3-BPG)	1.0633	0.9960	1.4021	2.1747	1.8841	1.7720	1.0595	1.0638	0.3559	0.2538	0.2148	0.0988
pyruvate	1.0719	0.6236	0.4910	0.3352	1.4589	1.3610	1.0068	1.6145	0.9836	2.0032	0.7456	2.2246
lactate	1.3268	0.8362	0.7292	0.6277	2.6118	1.9685	1.1590	1.3860	1.1252	1.5431	1.0443	1.6638
citrate	0.9248	0.9026	1.0539	1.0156	1.4429	1.5602	1.2492	1.3841	1.2093	1.1475	1.1147	1.0976
aconitate	0.8083	0.9314	1.2357	1.0678	1.1709	1.4485	1.3817	1.4835	1.4687	1.1886	1.5438	1.4458
succinate	0.9980	0.8435	0.7550	0.5561	1.8648	1.8684	1.0478	1.2421	1.3070	1.7311	1.0554	1.8978
fumarate	0.9338	1.0088	0.7889	0.8187	2.2378	2.3965	1.3569	1.3450	1.0111	1.2817	0.8542	1.0434
malate	0.8495	0.9233	0.7526	0.5330	2.2605	2.6610	1.3738	1.4880	1.0398	1.3817	0.7746	1.4533
succinyl CoA	0.9336	0.7717	0.9531	0.8903	0.5327	0.5706	1.4152	1.8339	1.5923	1.6707	1.7759	1.9947

Supplementary Table S2. Patients and liver biopsy data.

	Gender	BMI	HCV Blood IU/mL (Log10)	HCV Liver IU/mg (Log10)	Glucose (mmol/L)	Insulin (mIU /L)	C-peptide (pg/ml)	HOMA -IR	*HDAC9* (fold)	*PEPCK* (fold)	PEPCK (mU/mg) protein)	*PGC-1α* (fold)	*L-PGC-1α* (fold)	*CREB* (fold)
1	F	23.48	N	N	4.30	8.06	1002.07	1.54	1.00	1.00	5.90	1.00	1.00	1.08
2	F	30.86	N	N	5.12	7.26	863.71	1.65	1.25	1.12	3.10	1.13	1.03	1.59
3	F	20.73	N	N	3.89	8.34	1169.29	1.44	1.74	0.74	6.70	1.05	1.04	1.74
4	F	35.06	N	N	4.90	7.20	920.46	1.57	0.65	0.84	3.50	0.73	0.63	1.12
5	F	26.68	N	N	5.02	8.16	1003.40	1.82	0.54	1.15	4.70	0.97	1.01	1.00
6	M	29.02	N	N	4.46	8.55	1125.90	1.69	1.05	1.22	3.90	0.91	0.91	0.92
7	M	34.20	N	N	4.29	13.38	1209.80	2.55	0.69	0.77	5.00	0.70	0.70	0.81
8	M	24.24	N	N	4.17	10.19	1019.04	1.89	0.57	0.85	4.40	0.62	0.62	0.72
9	M	22.18	N	N	4.05	6.40	960.74	1.15	1.21	1.05	3.20	1.15	1.15	0.75
10	M	22.68	N	N	3.84	10.89	1488.78	1.86	1.37	1.23	5.10	1.28	1.28	N
11	M	N	N	N	4.2	N	N	N	1.00	N	N	0.96	0.73	0.91
12	F	N	N	N	5.5	N	N	N	0.94	N	N	1.13	1.00	0.44
13	M	N	N	N	4.7	N	N	N	1.49	N	N	1.20	1.21	1.01
14	F	N	N	N	4.1	N	N	N	0.81	N	N	1.000	0.49	0.55
15	F	N	N	N	5.3	N	N	N	1.14	N	N	1.15	1.12	0.81
16	F	24.24	6.53	8.12	5.76	11.68	1634.50	2.99	8.34	3.34	13.00	3.69	2.47	N
17	F	27.06	6.23	8.23	6.70	11.85	1573.61	3.53	7.85	2.85	12.30	2.60	2.21	N
18	F	24.22	5.75	8.39	10.60	12.03	1823.76	5.66	12.46	4.01	21.80	4.53	3.52	N
19	F	28.89	5.87	8.65	5.30	15.70	1388.30	3.67	6.79	2.89	17.60	2.88	2.97	N
20	F	26.95	6.08	8.04	5.80	25.25	2569.92	6.52	9.47	3.46	19.70	3.24	2.43	N
21	F	23.53	6.59	8.19	4.00	15.21	1543.86	2.71	6.66	2.99	12.40	2.35	2.31	N
22	M	24.26	5.63	8.63	5.20	12.42	1304.18	2.90	8.74	4.00	9.90	2.78	2.58	N
23	M	20.28	5.39	8.42	5.40	19.66	2001.65	4.71	10.48	3.37	15.30	3.13	2.95	N
24	M	24.22	6.42	8.00	4.07	10.41	2006.37	1.88	6.77	3.01	8.70	2.38	2.66	N
25	M	20.83	6.03	8.14	6.40	28.82	3399.21	8.15	14.38	5.01	14.30	5.92	3.21	N
26	M	25.61	5.81	8.38	6.10	9.83	1483.26	2.67	9.23	3.00	9.60	3.88	2.75	N
27	M	24.80	6.68	8.19	4.10	8.63	1073.24	1.58	7.41	2.89	14.50	2.51	2.06	N
28	F	24.49	6.41	8.16	4.60	10.81	952.62	2.22	8.55	3.42	13.00	2.74	2.37	N
29	M	24.49	6.58	8.10	4.00	13.60	1345.64	2.39	7.82	2.55	12.30	2.19	1.89	N
30	M	21.13	6.61	8.33	7.00	28.99	4248.51	9.07	11.79	4.32	21.80	4.26	2.25	N
31	F	17.96	7.00	8.64	5.70	16.07	1997.16	4.05	11.43	2.56	17.60	4.23	3.23	N
32	F	23.81	6.67	8.75	5.50	16.07	1779.60	3.90	6.65	3.67	19.70	2.35	4.15	N
33	F	23.44	6.41	8.09	6.70	22.72	3638.61	6.78	14.76	3.06	6.30	6.00	3.92	N
34	M	18.37	6.51	8.43	4.30	17.24	1771.91	3.26	6.44	4.00	7.40	2.31	1.30	N
35	F	23.26	6.65	7.80	5.00	9.52	1143.97	2.12	5.32	3.37	6.60	3.08	2.81	N
36	M	18.37	6.53	8.13	5.40	15.52	2980.46	3.76	9.77	3.01	11.10	3.99	2.96	N
37	M	23.74	5.75	8.37	5.10	8.97	1955.33	2.04	6.67	3.01	7.90	2.36	1.35	N
38	F	20.55	5.87	7.00	9.50	18.22	1598.91	7.73	5.49	2.88	8.40	1.12	1.55	N
39	F	22.89	4.83	7.04	4.60	11.22	887.11	2.30	4.31	1.22	6.30	0.88	1.28	N
40	M	22.49	6.68	8.30	5.50	14.46	1239.89	3.53	8.87	2.13	9.40	1.80	2.84	N
41	M	31.11	6.99	8.37	7.50	6.09	1281.86	2.05	7.96	1.98	10.20	1.62	2.65	N
42	M	27.34	6.89	8.17	6.50	35.42	3220.94	10.28	10.45	4.55	11.30	4.12	4.34	N
43	M	22.15	6.37	8.28	7.00	25.22	2383.44	7.91	9.96	3.14	9.60	3.02	1.39	N
44	M	20.76	5.08	8.14	7.10	21.90	2896.86	6.91	11.23	5.22	8.00	4.28	3.19	N
45	M	20.01	4.99	7.87	5.30	6.80	869.48	1.60	8.74	3.22	5.50	2.78	2.60	N
46	M	25.39	6.74	8.93	6.30	15.54	1833.70	4.37	5.34	2.78	7.90	1.85	1.50	N
47	M	29.41	4.89	6.30	6.60	22.14	2274.35	6.47	5.44	2.02	7.10	1.54	1.50	N
48	M	19.38	4.94	6.37	4.80	28.99	3325.84	6.21	6.67	1.98	6.70	1.36	1.34	N
49	M	27.43	5.08	7.39	7.20	23.45	2691.70	7.46	5.49	3.15	6.60	1.46	2.55	N
50	M	18.34	5.03	7.65	5.60	13.69	1836.55	3.39	7.23	4.55	8.00	3.47	2.91	N
51	M	27.68	4.98	6.14	5.80	20.49	1907.86	5.31	4.31	3.56	5.90	1.88	1.76	N
52	M	19.84	5.16	7.26	7.50	15.68	1304.51	5.22	8.45	4.32	6.20	3.72	2.70	N
53	M	24.49	5.20	6.38	6.40	14.56	1112.68	4.12	4.41	3.41	7.40	2.90	1.81	N

Supplementary Table S3. Primers for qPCR and ChIp and sequences of shRNA used in this study

Strand/Gene	Primer	Sequence(5'-3')
PGC-1α	PGC1F	TGAAAAAGCTTGACTGGCG
	PGC1R	AAGATCTGGGCAAAGAGGC
L-PGC-1α	LPGC1F	TCCCTCTGTTGCCTTTGTG
	LPGC1R	TAACCCCATGCCATCCAT
CREB	CREBF	ATTCACAGGAGTCAGTGGATAGT
	CREBR	CACCGTTACAGTGGTGATGG
CREB5	CREB5F	AAAGACTGCCCAATAACAGCC
	CREB5R	AAGCTGGGACAGGACTAGCA
HCV	HCVF	CCCTATCAGGCAGTACCACAAGG
	HCVR	ACCTGCCCCTAATAGGGGCG
Actin	ActinF	GAAATCGTGCGTGACATTAA
	ActinR	AAGGAAGGCTGGAAGAGTG
CREB-CHIP-1	CREBCF1	CCCCGCCTTTCTCCAGACAACA
	CREBCR1	GTAGAAAACAGGCTGAGGAAAG
CREB-CHIP2	CREBCF2	TCTTTAGGAAGCAGAGAGGGTT
	CREBCR2	GGAAAGGTTGAGAGTAACAGAA
GR-CHIP-1	GRCF1	ATGTGACTCCAACATGCTTTTG
	GRCR1	CAAAAGCATGTTGGAGTCACAT
GR-CHIP-2	GRCF2	TGTCAAAGAGCATGTCTCTGGC
	GRCR2	GAATTATCATTGTGCGTGGCAC
GR-CHIP-3	GRCF3	CTTATCTACGGAGGATATGTTC
	GRCR3	TGAGTTATTGTTAATCTCTTGC

Oligo name	Sequence (sense strand)
shHDAC9-1	CCGGCAAACTGCTTTCGAAATCTATCTCGAGATAGATTTCGAAAG
shHDAC9-2	CCGGGAGCAGTTAATAGGCTTTAAACTCGAGTTTAAAGCCTATTA
